# Antioxidant Status, Antidiabetic Properties and Effects on Caco-2 Cells of Colored and Non-Colored Enriched Extracts of Sweet Cherry Fruits

**DOI:** 10.3390/nu10111688

**Published:** 2018-11-05

**Authors:** Ana C. Gonçalves, Márcio Rodrigues, Adriana O. Santos, Gilberto Alves, Luís R. Silva

**Affiliations:** 1 CICS-UBI—Health Sciences Research Centre, University of Beira Interior, 6201-506 Covilhã, Portugal; anacarolinagoncalves@sapo.pt (A.C.G.); marciorodrigues@fcsaude.ubi.pt (M.R.); aos@ubi.pt (A.O.S.); gilberto@fcsaude.ubi.pt (G.A.); 2 UDI-IPG, Research Unit for Inland Development, Polytechnic Institute of Guarda, 6300-749 Guarda, Portugal

**Keywords:** sweet cherry, anthocyanins, non-colored phenolics, antioxidant activity, erythrocytes protection, Caco-2 cells

## Abstract

This study aimed to compare three different extracts of *Saco* sweet cherry, namely the non-colored fraction, colored fraction, and total extract concerning phenolic composition, antioxidant and antidiabetic potential, and erythrocytes’ protection and effects on Caco-2 cells. Twenty-two phenolic compounds were identified using high-performance liquid chromatography with diode-array detection. Hydroxycinnamic acids were the most predominant in both the non-colored fraction and total extract, while cyanidin-3-*O*-rutinoside was the main anthocyanin found in the colored fraction. The total extract was the most effective against 1,1-diphenyl-2-picrylhydrazyl, nitric oxide, and superoxide radicals, and in the inhibition of α-glucosidase enzyme. The colored fraction revealed the best activity against hemoglobin oxidation and hemolysis. Regarding to Caco-2 cells, the colored extract exhibited the highest cytotoxic effects, while the total extract was the most efficient in protecting these cells against oxidative damage induced by *tert*-butyl hydroperoxide.

## 1. Introduction

Phenolic compounds are widely distributed in nature and present strong antioxidant properties [1]. It is believed that their presence in the daily diet exerts a beneficial effect on human health, being associated with the decrease of oxidative stress-related disorders’ occurrence [1,2]. Furthermore, phenolic compounds reduce the rate of oxidative processes by acting as reducing agents, hydrogen donors, singlet oxygen quenchers, and metal chelators, inhibiting the propagation of oxidizing chain reactions caused by free radicals and protecting the human body against oxidative damage [3,4].

Recently, special attention has been paid to the use of plants and fruit extracts in the cosmetic, food, and pharmaceutical industries due to their richness in phenolic compounds [5,6]. The extractions to obtain fractions rich in bioactive substances are preferentially carried out using water–alcohol mixtures, often including ethanol or methanol as extraction solvents, given their affinity with both lipophilic and hydrophilic bioactive molecules. Ethanol is the most commonly used solvent because it is economical, reusable, and unlike methanol, is non-toxic [5].

Extracts of sweet cherry (*Prunus avium* Linnaeus (L.)) have been subjected to several studies due to their properties as health promoters [7,8,9]. Therefore, sweet cherry berries may have a great potential in the management of obesity, diabetes, and related comorbidities [10]. Although these fruits are preferably consumed fresh, they can also be commercialized in processed products such as frozen, canned, wine or concentrate juices, jellies, jams, and dried forms [2]. They are rich in several non-colored (chlorogenic acids, flavan-3-ols, and flavonols) and colored (mainly cyanidin-3-*O*-rutinoside) phenolic compounds, whose levels are regulated by genotype, fruit maturity, climatic conditions, and storage conditions [6,7].

The largest European cherry-producing countries are Poland, Spain, Italy, Greece, Hungary, and Germany [11]. In Portugal, around 15 thousand tons of cherries are produced per year. Most of them are collected from the Fundão region, with *Saco* being one of the most important and oldest cultivars [7]. Previous works with *Saco* proved their large array of health benefits, namely antioxidant effects [6,7,9], anticancer activity against human cancer cells from the colon (HT-29 and HCT-15) and stomach (MKN45) [6,9], antidiabetic properties, and capacity to confer protection to human erythrocytes against hemoglobin oxidation and hemolysis [7].

Considering the above-mentioned reasons, the aim of this work was to improve the knowledge on the phenolic profile of the total extract, and colored and non-colored fractions of *Saco* sweet cherry from the Fundão region (Portugal). Furthermore, and knowing that colored and non-colored compounds can interact with each other in synergistic, additive, and antagonistic ways, we also evaluated their antioxidant potential against 1,1-diphenyl-2-picrylhydrazyl (DPPH), nitric oxide (^•^NO), and superoxide (O_2_^•−^) radicals. The capacity of the extracts to inhibit *α*-glucosidase enzyme was also determined, as well as the protection afforded against induced oxidative damage in human erythrocytes. Additionally, the cytotoxic properties of the extracts were assessed for the first time against human colon carcinoma cells (Caco-2) under quiescent conditions concerning their antiproliferative activity and toxicology responses when subjected to oxidative stress induced by *tert*-butyl hydroperoxide (*t*-BHP).

## 2. Materials and Methods

### 2.1. Chemicals and Reagents

All chemicals used were of analytical grade. Cyanidin-3-*O*-glucoside, cyanidin-3-*O*-rutinoside, pelargonidin-3-*O*-rutinoside, and peonidin-3-*O*-rutinoside were from Extrasynthese (Genay, France). The other phenolics and DPPH, *β*-nicotinamide adenine dinucleotide (NADH), phenazine methosulfate (PMS), nitrotetrazolium blue chloride (NBT), *α*-glucosidase from *Saccharomyces cerevisiae* (type I, lyophilized powder), trypan blue, 2,2′-azobis (2-ethylpropionamidine) dihydrochloride (AAPH), *tert*-butyl hydroperoxide (*t*-BHP), high-glucose Dulbecco´s Modified Eagle Medium (DMEM), fetal bovine serum (FBS), antibiotics (10,000 U/mL penicillin, 10,000 mg/mL streptomycin), trypsin-ethylenediaminetetraacetic acid (trypsin-EDTA) solution, 3-(4,5-dimethylthiazol-2-yl)-2,5-diphenyltetrazolium bromide (MTT), dimethyl sulfoxide (DMSO), pyruvate, propidium iodide (PI), bovine serum albumin (BSA), and Ribonuclease A (RNase) were purchased from Sigma-Aldrich (St. Louis, MO, USA). *N*-(1-naphthyl)ethylenediamine dihydrochloride, sulfanilamide, 4-nitrophenyl-alpha-D-glucopyranoside (pNPG), and sodium nitroprusside dihydrate (SNP) were obtained from Alfa Aesar (Karlsruhe, Germany). Methanol and acetonitrile were acquired from Fisher Chemical (Leicestershire, Glenfield, United Kingdom). Water was deionized using a Milli-Q water purification system (Millipore Ibérica, S.A.U., Madrid, Spain). Caco-2 cells were from the American Type Culture Collection (ATCC; Manassas, VA, USA).

### 2.2. Cherry Samples

One kg of *Saco* sweet cherry cultivar was collected by hand from an orchard located in Fundão region (Portugal) at the same stage of ripeness and age (three years old), during June of 2016. The cherries were transported to the laboratory of Health Sciences Research Centre (CICS) of the University of Beira Interior (Covilhã, Portugal). The pits were removed, and the pulp was immediately frozen with liquid nitrogen and maintained at −20 °C. Then, the pulp was lyophilized, powdered (mean particle size lower than 910 μm), and separated into three aliquots and used for the preparation of the extracts.

### 2.3. Phenolic Compounds

#### 2.3.1. Extraction

Phenolic compounds of *Saco* samples were extracted according to Gonçalves et al. [7]. Briefly, 1 g of dried Saco was stirred at 300 rpm with ethanol (70:30) for 2 h, and then centrifugated at 4000 rpm for 10 min. After that, the supernatant was evaporated under reduced pressure at 30 °C and the resulting extract was dissolved with 50 mL of deionized water and placed into a C18 solid-phase extraction (SPE) column (70 mL/10,000 mg; Macherey–Nagel, Düren, Germany) previously conditioned with ethyl acetate, ethanol, and 0.01 mol/L HCl. After passing the sample, the column was washed with 3 mL of 0.01 mol/L HCl. Afterward the fraction I (non-colored phenolics) was eluted with 20 mL of ethyl acetate and placed in an erlenmeyer, while the fraction II (anthocyanins) was eluted with 40 mL of ethanol containing 0.1% HCl and placed in another erlenmeyer. To obtain the fraction III (total extract), another C18 solid-phase extraction was performed, with this one being eluted with 40 mL of ethanol containing 0.1% HCl. Finally, each fraction was evaporated to complete dryness and frozen at −20 °C until analysis. The extraction yields of non-colored fraction, colored fraction, and total extract were 8.5 ± 0.02%, 3.6 ± 0.005%, and 13.0 ± 0.01%, respectively.

#### 2.3.2. HPLC-DAD Analysis

Twenty microliters of each sample were analyzed on a LC model Agilent 1260 system (Agilent, Santa Clara, CA, USA) using a Nucleosil^®^ 100-5 C18 column (25.0 cm × 0.46 cm; 5 μm particle size waters; Macherey-Nagel, Düren, Germany), based on a previously published method [7]. Detection was achieved with an Agilent 1260 Infinity DAD using the ChemStation software supplied by Agilent Technologies (Waldbronn, Germany). The non-colored fraction was re-dissolved with 4 mL of methanol, while the colored fraction was dissolved with 20 mL of acidified water with pH 3.0. The total extract was again eluted using an SPE column to separate the non-colored and colored phenolics (anthocyanins) for identification and quantification. After dissolution, each fraction was filtered using a 0.45 μm polytetrafluoroethylene membrane (Millipore, Bedford, MA, USA) prior analysis.

The detection and quantification of anthocyanins and non-colored phenolic compounds were performed according to Gonçalves et al. [7]. The compounds of each extract were identified by comparing their retention times and ultraviolet–visible spectra in the 200–600 nm range with the library of spectra previously compiled by the authors. Anthocyanins were detected at 500 nm. Flavan-3-ols and hydroxybenzoic acids were detected at 280 nm, hydroxycinnamic acids at 320 nm, and flavonols at 350 nm. The unknown compounds 1–3 were identified as cyanidin-3-*O*-rutinoside. The hydroxybenzoic acid derivative 1 was identified as *p*-hydroxybenzoic acid. The 3-*O*-caffeoylquinic acid and hydroxycinnamic acid derivatives 1 and 2 were identified as 5-*O*-caffeoylquinic acid. *p*-Coumaric acid derivatives 1–5 were identified as *p*-coumaric acid. The total phenolics and total anthocyanins (∑) were the result of the sum of each determined compound belonged to non-colored phenolics or anthocyanins, respectively.

### 2.4. Biological Acitivity Evaluation

The evaluation of the biological potential of the non-colored fraction, colored fraction, and total extract was performed spectrophotometrically by in vitro microassays using 96-well plates. The absorbances were measured in a microplate reader Bio-Rad Xmark spectrophotometer (Bio-Rad Laboratories, Hercules, CA, USA).

#### 2.4.1. Antioxidant Capacity

##### DPPH

The capacity of non-colored, colored fractions, and total extract to act as free radical scavengers against DPPH^•^ was evaluated as previously reported [7].

##### Nitric Oxide

The activity of the dried extracts against ^•^NO was determined as previously described by Gonçalves et al. [7].

##### Superoxide Radical

The effect of cherry extracts on the O_2_^•−^-induced reduction of NBT was monitored at 562 nm. O_2_^•−^ was generated by the PMS-NADH-O_2_ system, as previously reported [12].

#### 2.4.2. α-Glucosidase Inhibitory Activity

The *α*-glucosidase inhibitory activity was determined at 405 nm, based on Ellman’s method, as previously described by Silva and Teixeira [12].

#### 2.4.3. Protective Effect in Human Erythrocytes against Oxidative Damage

Venous human blood was collected from randomized patients of Cova da Beira Hospital Centre (Covilhã, Portugal) by antecubital venipuncture into K_3_EDTA vacuum tubes. Erythrocytes were isolated based on the procedure previously described [13].

##### Inhibition of Hemoglobin Oxidation

The inhibition of hemoglobin (Hb) oxidation was evaluated by monitoring the effects of the three *Saco* extracts on the formation of methemoglobin after the reaction of oxyhemoglobin with peroxy radicals (ROO^•^) generated by AAPH [7,13,14]. The absorbance was read at 630 nm. Quercetin was used as a positive control. Three experiments were performed in triplicate for each extract.

##### Inhibition of Hemolysis

Peroxy radicals (ROO^•^s) were generated using AAPH and the prevention of ROO^•^-induced hemolysis of human erythrocytes was evaluated by monitoring the release of Hb after the membrane disruption caused by the hemolytic process according to the procedure described by Chisté et al. [13] and Gonçalves et al. [7]. The absorbance was obtained at 540 nm. Quercetin was used as a positive control. Three experiments were performed in triplicate for each extract.

### 2.5. Cell Culture Conditions and Treatments

Caco-2 cells were maintained in DMEM supplemented with 10% FBS and 2% of Pen/Strep. Cells were grown in 75 cm^2^ culture flasks at 37 °C in a humidified air incubator with 5% CO_2_. Once the cells reached 90–95% of confluence, they were washed twice with 10 mL of phosphate-buffered saline (PBS) and detached by gentle trypsinization (5 mL of trypsin-EDTA), and before the experiments, viable cells were counted and suitably diluted in the adequate complete culture medium (25,000 cells/mL). These cell culture conditions and procedures were common through all assays. For the several assays, cells were used between passages 44 and 60. After the trypsinization and count of the cells, 200 µL of the prepared cellular suspension (25,000 cells/mL) was seeded in 96-well plates and incubated for one day before carrying out the viability assays. Five concentrations in the range of 50–800 µg/mL of each extract were dissolved in medium containing 0.5% (*v*/*v*) DMSO. The final concentration of DMSO did not affect cellular viability (data not shown).

#### 2.5.1. Cytoprotection Assay

Preliminary assays were done to determine the appropriate concentration and exposure time to *t*-BHP in order to evaluate the activity of the cherry extracts (data not shown). The *t*-BHP dilutions were prepared with concentrations that ranged from 0.25 to 4 mM and cells were exposed for 2, 4, and 6 h. For the analysis of the obtained results, and to achieve a suitable viability decrease, the selected exposure conditions were a time of 6 h with *t*-BHP at 1 mM. Cells were seeded under the same conditions previously described, with and without *t*-BHP co-incubation. After cell incubation for 24 h, the medium was removed, and cells were treated with the extracts for 24 h, then the *t*-BHP was added to each well plate for 6 h. The difference of the assay without incubation was that after 24 h of incubation with extracts, the medium was completely removed, and 1 mM of *t*-BHP was added to each well for 6 h. Finally, the MTT and lactate dehydrogenase (LDH) assays and the cell cycle distribution were carried out to evaluate the effect of the extracts against the induced toxicity.

#### 2.5.2. MTT Cell Proliferation Assay

To determine the effect of extracts on Caco-2 cells, viability was assessed using an MTT assay after 24 h of exposure of each extract at different concentrations. Then, the medium was removed, and each well was washed with 200 µL of PBS. The metabolic activity of cells was evaluated via their capacity to reduce the yellow MTT (0.5 mg/mL in the appropriate serum-free medium) to a blue formazan product using 4 h of incubation at 37 °C. Then, the medium containing MTT was removed and the formazan crystals were dissolved in DMSO. The absorbance was read at 570 nm using a microplate reader Bio-Rad Xmark spectrophotometer. Cell proliferation values were expressed as percentages from the relative absorbance measured in the treated wells versus control wells [12,15]. A total of six independent experiments per extract were performed. Untreated cells were used as control.

#### 2.5.3. LDH Assay

The release of the stable cytosolic enzyme LDH into the media was spectrophotometrically determined at 340 nm, based on the conversion of pyruvate to lactate by LDH, using NADH as a cofactor [12]. The reaction mixture was composed of the extracts NADH and pyruvate. All solutions were prepared in PBS (pH 7.4). The medium of each well plate treated with the extracts was collected after 24 h of cellular exposure and the LDH was evaluated. A total of six independent experiments per extract were performed. Untreated cells were used as a control.

#### 2.5.4. Cell Cycle Distribution Analysis

The analysis of the cell cycle distribution of cells was determined through PI staining of fixed and permeabilized cells. Briefly, 2.4 mL of Caco-2 cells were seeded in 12 well-plates (cell density of 25,000 cells/mL) in complete culture medium. After 24 h, they were treated with 200, 400, and 800 µg/mL of each extract. Untreated cells were used as a negative control. At the end of 24 h of incubation, each well was washed with PBS and the cells were harvested using a trypsin treatment. The resulting cell suspension was kept on ice, pelleted via centrifugation, and resuspended in 450 μL of a cold solution of 0.5% BSA in PBS. The resulting cell suspension was kept on ice and then fixed by gently adding ice-cold 70% ethanol (−20 °C) with simultaneous vortexing. After 1 day at −20 °C, fixed cells were washed twice with PBS and resuspended in a solution of PI prepared in PBS/BSA 0.5% (50 μg/mL), passed through cell strainer filters (40 µm nylon, Falcon^®^, Life Sciences, Wiesbaden, Germany) and sequentially incubated with RNase at a final concentration of 7.1 μg/mL (stock solution in 50% glycerol, 10 mM Tris-HCl, pH 8) for 15 min in the dark. Stained cells were then acquired at a BD Biosciences FACSCalibur flow cytometer (San Jose, CA, USA), using the 488 nm laser. Data were analyzed using CellQuest™ Pro Software, version 5.1.1[M1] (BD Biosciences, San Jose, CA, USA). First, singlets were selected by creating a region on the Fluorescence channel 3 (FL3)-Width/FL3-Area contour plot. FL3 signal corresponds to a wavelength > 670 nm. Then, gated singlet events were plotted on the Forward Scatter (FSC)-Height/FL3-Area contour plots for correlation of size with PI-staining.

### 2.6. Statistical Analysis of Results

All data were recorded as mean ± standard deviation of triplicate determinations. The HPLC-DAD statistical phenolic comparison was made using the two-way ANOVA and the Bonferroni test. About to the biological potential, the statistical comparison was assured by the one-way ANOVA, and the means were classified by Tukey’s test at a 95% level of significance. To determine the correlation between the antioxidant activity methods and the contribution of the total phenols, Pearson’s correlation coefficients were calculated. Relative to the cellular-based assays, data from different groups were compared by two-way ANOVA followed by Dunnett’s test (LDH and MTT assays) as a *post*-*hoc* test. Values of *p* < 0.05 were considered statistically significant. All analyses were performed using Graph Pad Prism Version 6.01 (San Diego, CA, USA).

## 3. Results and Discussion

### 3.1. Phenolic Profile

The chromatographic analysis allowed the identification and quantification of a total of 22 phenolic compounds, including 2 hydroxybenzoic acid derivatives, 14 hydroxycinnamic acids, 1 flavonol, and 5 anthocyanins (Table 1 and Table 2). All of these compounds were previously described in *Saco* sweet cherry from Fundão (Portugal) [6,7]. As expected, the non-colored phenolics were only found in the total extract and non-colored fraction. Nevertheless, both extracts showed different quantitative composition (Table 1). The total contents of the non-colored phenolics in the total extract and non-colored fraction were 11,069.7 and 15,220.9 μg/g of dried extract, respectively (Table 1).

With respect to phenolic acids and derivatives, they corresponded to 69.8% and 99.7% of the total phenolic compounds determined in the total extract and non-colored fraction, respectively (Table 1). The main compounds were hydroxycinnamic acid derivative 4 and 3-*O*-caffeoylquinic acid, representing 17.9% and 9.4% in the total extract, and 25.6% and 13.4% in the non-colored fraction, respectively (Table 1). These results agreed with previous works, where the authors reported that the major compounds found in sweet cherries are hydroxycinnamic acids, mainly the 3-*O*-caffeoylquinic acid being the one that was also the most abundant compound found in *Saco* [6,7,9].

Additionally, one flavonol was detected and identified as quercetin, corresponding to 0.23% and 0.32% of total phenolic content in total extract and non-colored fraction, respectively. No flavan-3-ols (e.g., catechins) were detected. This fact was not surprising given that Gonçalves et al. [16] have already observed that catechin levels decreased in *Saco* cultivar during storage at room temperature (15 ± 5 °C) for 6 days.

With respect to anthocyanins, there were identified and quantified five compounds (Table 2). All of these colored compounds were previously reported in *Saco* sweet cherry [6,7].

The total amount of anthocyanins found in the total extract and colored fraction were 4740.7 and 19,214.5 μg/g of dried extract, respectively (Table 2). Despite the different amounts of each anthocyanin observed, both extracts revealed a similar profile, being the unique difference related to the presence of two unknown anthocyanins detected only in the total extract (Table 2). Cyanidin-3-*O*-rutinoside was the main one quantified in both extracts, representing 24.5% and 81.5% of the total compounds for the total extract and colored fraction, respectively, followed by cyanidin-3-*O*-glucoside. These data are in agreement with other previously reported works [6,7,10].

Comparatively with other cherries, tart cherries are richer in cyanidin-3-*O*-glucosylrutinoside (357.7 µg/g of dried extract), followed by cyanidin-3-*O*-rutinoside (226.1 µg/g of dried extract), and quercetin than sweet cherries (292.6 µg/g of dried extract) [17].

### 3.2. Antioxidant Capacity

Reactive species, including ^•^NO, O_2_^•−^, and ROO^•^, are products of normal cellular metabolism, playing crucial roles in signal transduction pathways, growth regulation, gene expression, and immune responses [4]. Normally, their production in the human body is balanced by antioxidants; however, when they are overproduced, they cause damage in DNA, lipids, and proteins, potentiating many human diseases, including cancer, diabetes, necrosis, neurological disorders, and cardiovascular illnesses [1].

**Figure 1:**
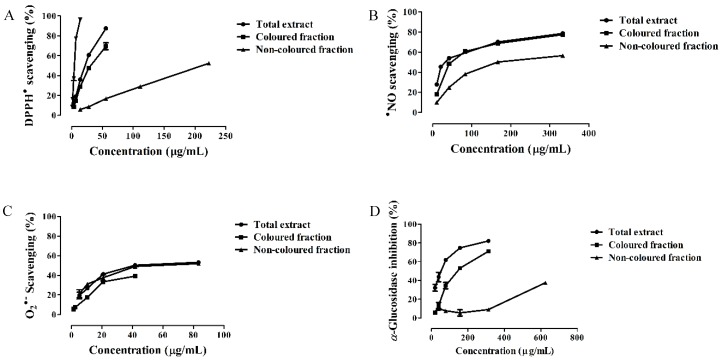
Antioxidant activity against (**A**) 1,1-diphenyl-2-picrylhydrazyl radical (DPPH^•^), (**B**) nitric oxide radical (^•^NO) and (**C**) superoxide radical (O_2_^•−^), and (**D**) α-glucosidase inhibition activity of *Saco* sweet cherry extracts.

1,1-Diphenyl-2-picrylhydrazyl (DPPH) assay is widely used to determine the antioxidant activity of single compounds and plant extracts. All extracts studied in this work revealed to be able to scavenge DPPH radical in a concentration-dependent manner. The total extract was the most active (IC_50_ = 21.88 ± 0.32 µg/mL), followed by the colored and non-colored fractions (IC_50_ = 31.39 ± 0.60 and 210.86 ± 0.86 µg/mL, respectively). However, all extracts were less active than ascorbic acid control (IC_50_ = 4.57 ± 0.16 µg/mL) (Figure 1A and Table 3). Since phenolics are good hydrogen and electron donors and interact with each other rising the antioxidant ability, it was expected that the total extract, which has both colored and non-colored compounds, would be the most active [18].

The result obtained from the total extract is in accordance with our previous work relative to the *Saco* sweet cherry DPPH scavenging ability (IC_50_ = 16.24 ± 0.46 µg/mL) [7]. Considering other red-fruit extracts, the three extracts showed more potential than anthocyanin-enriched fraction of Romina strawberry fruits (IC_50_ = 1590 ± 3.54 µg/mL), while the colored fraction of *Saco* proved to have similar activity to whole methanolic extract of strawberries (IC_50_ = 30.29 ± 0.18 µg/mL) [19]. In addition, our total and colored extracts revealed to be more active than crude, petroleum ether, chloroform, ethyl acetate, and butanol fractions of myrtle fruits (*Myrtus communis*) (IC_50_ = 130.44 ± 12.1, 108.3 ± 10.2, 111.14 ± 11.1, 85.6 ± 5.7, and 84.42 ± 1.8 µg/mL, respectively) [20].

The antioxidant capacity of the extracts also was evaluated against ^•^NO and O_2_^•−^. Both radicals are present in the human body. While NO is normally produced in the organism as a second messenger and as a part of immune response, its reaction with O_2_^•−^ leads to the production of more toxic free radical species, such as peroxynitrite and ROO^•^, increasing deleterious effects in cells [1]. Presently, there is much evidence about the beneficial effects of phenolic-rich extracts on ^•^NO and O_2_^•−^ species’ elimination [7,12].

In this study, all *Saco* extracts displayed a great capacity to capture ^•^NO in a concentration-dependent manner (Figure 1B and Table 3). The total extract was the most active (IC_50_ = 33.72 ± 0.89 µg/mL), followed by the colored and non-colored fractions (IC_50_ = 47.44 ± 0.67 and 167.96 ± 0.92 µg/mL, respectively) (Figure 1B and Table 3). All extracts showed a better ability to scavenge ^•^NO than ascorbic acid control (IC_50_ = 179.69 ± 2.06 µg/mL). The total extract was the most effective, given the fact that the natural combination of both non-colored phenolics and anthocyanins increases the capture of radical species though electron delocalization after hydrogen donation by the hydroxyl (OH) groups, diminishing the concentration of these undesirable species [4].

Indeed, the capacity of sweet cherries to scavenge ^•^NO was previously reported by Jacob et al. [21]. The authors verified a decrease in NO levels in ten healthy women 3 h after the consumption of 280 g of Bing sweet cherries. Relatively to other red-fruits, our extracts showed better activity than ethanolic (IC_50_ = 1600 ± 0.03 µg/mL) and aqueous extracts (IC_50_ = 1500 ± 0.02 µg/mL) of blueberry fruits (*Vaccinium corymbosum* L.) [1]. In addition, both the total extract and colored fraction proved to be more active than ethanolic strawberry extracts (IC_50_ = 118 ± 45.2 µg/mL) [22]. In comparison with ethanolic grape extracts, grapes displayed a better ability at neutralizing ^•^NO (IC_50_ = 5.1 ± 0.2 µg/mL) [22].

With respect to the ability of *Saco* extracts to scavenge O_2_^•−^, all of them showed activity in a concentration-dependent effect. The results obtained from total and non-colored extracts were IC_50_ = 41.68 ± 0.72 and 69.40 ± 1.22 µg/mL, respectively (Figure 1C and Table 3). The colored fraction revealed the weaker activity (IC_25_ = 16.58 ± 0.27 µg/mL). Even so, all the three extracts demonstrated to be less active than ascorbic acid control (IC_50_ = 28.87 ± 0.38 µg/mL). The total extract was the most active. The presence of both colored and non-colored phenolics increases and diversifies their interactions, and intensifies the antioxidant activity [1].

The scavenging ability of sweet cherry fruits against O_2_^•−^ was already demonstrated by Prior et al. [23]. With respect to other red fruits, our total extract exhibited similar activity to the vitamin C rich fraction of bilberry fruits (*Vaccinium myrtillus* L.) (IC_50_ = 49 µg/mL), but less efficiency than their flavonoid (IC_50_ = 21 µg/mL) and phenolic acid (IC_50_ = 15 µg/mL) rich fractions [24]. Moreover, our total extract and non-colored fraction proved to have higher ability to scavenge these species when compared with ethanolic extracts of strawberry fruits (IC_50_ = 72.5 µg/mL), but less activity than those of acetone (IC_50_ = 9.7 µg/mL). Furthermore, our total extract showed similar activity to strawberry ethyl acetate extracts (IC_50_ = 40.1 µg/mL) [25].

Our work is another report that supports the strong connection between the antioxidant capacity and the phenolic content. It is well-known that phenolic compounds can easily capture free radical species and chelate metal ions due to their acid moiety and OH groups [18]. The phenolic OH groups on the B-ring easily donate hydrogen atoms or an electron to radicals, stabilizing them [1]. Additionally, the OH groups present in the unsaturated C-ring and the double bond on this ring also enhance the antioxidant capacity [4]. Following this same order of context, it is important to emphasize the presence of cyanidin-3-*O*-rutinoside in both the total extract and colored fraction. This anthocyanin presents several OH groups in its structure, being one of the phenolics more responsible for the biological activity of the fruits [26]. Furthermore, both colored and non-colored phenolics interact with each other, through additive and synergistic combinations, conferring to sweet cherries great antioxidant effects [1,18].

In fact, by Pearson’s test, positive correlations were found between the antioxidant tests and the total amount of phenolic compounds. High correlations (*r* > 0.9013) were obtained between the majority of the non-colored phenolics, anthocyanins, and antioxidant activities measured by DPPH^•^, ^•^NO and O_2_^•−^ assays. Even so, we found a negative correlation between the O_2_^•−^ scavenging test, cyanidin-3-*O*-glucoside (*r* = −0.9818), and cyanidin-3-*O*-rutinoside (*r* = −0.9818), and between DPPH^•^ and ^•^NO antioxidant assays, and pelargonidin-3-*O*-rutinoside (*r* = −0.9241 and −0.9660, respectively). Finally, it is important to remember the possible presence of other non-determined reducing compounds, such as organic acids, carotenoids, and volatile compounds, which may also contribute to increasing the antioxidant potential, and that were not determined in this work.

### 3.3. α-Glucosidase Inhibitory Activity

Diabetes mellitus is an epidemic metabolic disease, characterized by chronic hyperglycemia and glucose intolerance caused by defects in insulin hormone that affects millions of people worldwide [2]. One therapeutic approach is to restore blood glucose levels as close to normal as possible via the inhibition of carbohydrate hydrolyzing-enzymes, such as α-glucosidase [27]. Over the years, many phenolic compounds present in plants have shown to be capable of inhibiting the action of α-glucosidase, thereby delaying glucose uptake [7,27].

In our study, all *Saco* extracts tested proved to have the capacity to inhibit the α-glucosidase enzyme as a concentration-dependent effect. The total extract showed the most effectiveness (IC_50_ = 53.15 ± 1.32 µg/mL), followed by the colored (IC_50_ = 142.02 ± 1.17 µg/mL) and non-colored fractions (IC_25_ value of 456.19 ± 3.74 µg/mL) (Figure 1D and Table 3). The obtained IC_50_ values were much lower than the acarbose control (IC_50_ = 389.89 ± 4.01 µg/mL), one of the therapeutic drugs most recommended to treat type 2 diabetes but whose use is limited since it causes gastrointestinal problems such as diarrhea, flatulence, and intestinal pain [7]. Both colored and non-colored phenolics interact among all and with the substrate of the enzyme and create bonds with this one, thus interfering with its action and consequently preventing the carbohydrate digestion, as well as protecting pancreatic β-cells from oxidative stress levels, contributing to their normal functioning [28].

The antidiabetic properties of sweet cherries are already known [7,29,30]. Lachin [30] reported that diabetic rats fed with 200 mg of cherry extract per kg of body weight for 30 days showed reduced blood glucose and urinary microalbumin levels, proving that the ingestion of these fruits can protect pancreatic β-cells and retard glucose absorption. Besides, Cao et al. [29] revealed that fractionated extracts of Black Pearl sweet cherry cultivar rich in anthocyanins, hydroxycinnamic acids, and flavonols also demonstrated antidiabetic properties by promoting cellular glucose consumption in HepG2 cells.

Comparing to the antidiabetic potential of other fruit extracts, our total extract and colored fraction exhibited a better capacity to inhibit α-glucosidase enzyme than dried crude acetone extracts of *Ficus lutea* and *Ficus sycomorus* (IC_50_ = 290 ± 111 and 217 ± 69 µg/mL, respectively) [31].

The positive results obtained with *Saco* extracts to inhibit α-glucosidase action are largely due to their phenolic constituents. In these ones, the unsaturated C-ring, the linkage of the B-ring at the position 3, the OH substitution on the B-ring, and the presence of either 3-OH and 4-CO groups enhance their ability to inhibit α-glucosidase [3].

In addition, they interact not only synergistically and additively between them and with other compounds, but also in both competitive and non-competitive ways with the substrate of the enzyme, increasing the antidiabetic potential of this fruit [3,7]. Also, phenolics can bond with digestive enzymes through hydrophobic association, inhibiting the action of them [32]. For these reasons, both colored and non-colored phenolic compounds can promote the insulin production and the proliferation of pancreatic β-cells and can interfere with glucose homeostasis by modifying independent mechanisms and increasing insulin receptor-dependents (such as peroxisome proliferator-activated receptor gamma, PPARγ), thus exerting antidiabetic actions [33].

In our study, it is possible to state that non-colored compounds have practically no significant and notorious effects on the inhibition of α-glucosidase, whilst anthocyanins showed great effectiveness. These results were expected, since previous studies reported that the colored compounds are the main compounds responsible for the antidiabetic capacities revealed by cherries, mainly due to their high antioxidant abilities (that are enhanced by the presence of the 3-OH group and the OH substitution on the B-ring in anthocyanins) that allow the protection of insulin mechanisms against oxidative damage and offer capacity to inhibit intestinal enzymes [3,28,34]. To reinforce this fact, the Pearson’s test was performed and there was a high correlation between the α-glucosidase inhibitory assay and the total amount of anthocyanins (*r* = 0.9929).

Additionally, the presence of rutinose in the 3-*O*-position of cyanidin, which already has many OH groups, reinforces this health-promoting property [28]. Indeed, the main colored compound present in sweet cherries, that is cyanidin-3-*O*-rutinoside, already has been demonstrated to have a significant inhibitory effect in α-glucosidase activity in a concentration-dependent manner by competing with glucose for the binding site on sodium-dependent glucose transporter (SGLT) 1, and consequently delaying the absorption of glucose [3,35]. More recently, Adisakwattana et al. [35] reported that cyanidin and its glycosides also create covalent and/or non-covalent interactions between their OH groups and the polar groups (amide, guanidine, peptide, amino, and carboxyl groups) of amino acids in the active site of carbohydrate digestive enzymes, stopping their action. The same work also revealed that a single oral administration of cyanidin-3-*O*-rutinoside (100 and 300 mg/kg) significantly decreases plasma glucose levels in rats at 30, 60, and 90 min after maltose and sucrose administration. Furthermore, cyanidin-3-*O*-glucoside also promotes the release of insulin by pancreatic β-cells and glucose uptake by enhancing glucose transporter GLUT 4 [33].

Non-colored compounds can also interfere with the activity of digestive enzymes and prevent insulin resistance events [34]. Particularly chlorogenic acids already showed to be able to increase GLUT 4 expression via the phosphatidylinositol 3-kinase (PI3K)-independent pathway, as well as to suppress hepatic gluconeogenesis through the inhibition of glucose 6-phosphatase activity, and to inhibit Na^+^-dependent glucose transporters SGLT 1 and SGLT 2 [34]. Caffeic acid also showed ability to enhance glucose-stimulated insulin secretion in mice pancreatic islets at concentrations from 10^–10^ to 10^−6^ M and the expression of key insulin regulatory genes INS1, INS2, PDX1, INSR, IRS1, MAFB, and GLUT2 [36].

### 3.4. Protective Effects of Saco Extracts against ROO• in Human Blood Samples

Erythrocytes are highly susceptible to being attacked by reactive species due to their content in unsaturated fatty acids, oxygen, hemoglobin, and in transition metals such as copper and iron [37]. In addition, they are more frequently exposed to oxygen than other body tissues and so they are more vulnerable to oxidative damage, causing the oxidation of lipids and proteins in the cell membrane, and thus inducing hemolysis [38]. Previous studies already have shown that phenolics present in plants offer therapeutic benefits against oxidative damage in erythrocytes [7,38,39].

**Figure 2:**
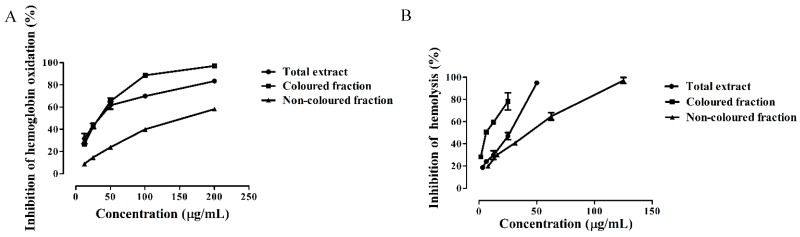
Inhibition of (**A**) hemoglobin oxidation and (**B**) hemolysis by *Saco* sweet cherry extracts.

Figure 2A shows the protective effects of *Saco* extracts to avoid hemoglobin oxidation in a concentration-dependent manner. To the best of our knowledge, this is the first study about the capacity of colored and non-colored rich fractions of sweet cherries to prevent hemoglobin oxidation. The colored fraction and the total extract were the most active and displayed similar activity (IC_50_ = 33.86 ± 0.70 and 34.29 ± 0.88 µg/mL, respectively) (Figure 2A and Table 3); however, they were seven times less active than quercetin control (IC_50_ = 4.38 ± 0.42 µg/mL) analyzed in the same conditions. The non-colored fraction showed an IC_50_ value of 155.13 ± 1.45 µg/mL (Figure 2A and Table 3*).*

As far as we know, few studies exist on the ability of fruit extracts to prevent the oxidation of hemoglobin. Previously, we have reported that the total extract of *Saco* possesses the capacity to prevent hemoglobin oxidation [7]. Hydrophilic extracts of murici fruits (*Byrsonima crassifolia*) also showed this property, revealing an IC_50_ of 271 ± 44 µg/mL [14], being less active than *Saco* extracts.

The capacity of phenolic compounds to inhibit oxidative damage is closely associated with the strong antioxidant capacity exhibited by themselves [18]. Both colored and non-colored phenolics can penetrate into the erythrocytes’ membrane due to their lipophilic character, causing a reduction in its fluidity and stability, and thus preventing the propagation of these harmful radicals [37]. The biological activity of the total extract and colored fraction is enhanced by the presence of catechol rings and OH groups, principally in cyanidin-3-*O*-rutinoside, increasing the potential of both extracts [40], as well as the ability to inhibit the oxidation of hemoglobin by oxidizing the heme iron of erythrocytes [41]. In this way, they prevent enzymatic reactions and consequently oxidative events.

Another experiment was conducted in order to evaluate the capacity of *Saco* extracts to prevent hemolysis. The addition to 2,2′-azobis (2-ethylpropionamidine) dihydrochloride (AAPH) generated ROO^•^ that is capable of attacking the membrane of erythrocytes from the outside, promoting hemolysis [37]. As far as we know, this is the first report concerning lysis prevention by both colored and non-colored fractions of sweet cherry fruits. The colored fraction (IC_50_ = 9.44 ± 0.48 µg/mL) showed the best anti-hemolytic protection, followed by the total extract (IC_50_ = 28.71 ± 0.73 µg/mL) and non-colored fraction (IC_50_ = 48.31 ± 1.07 µg/mL) (Figure 2B and Table 3). All extracts exhibited less activity than quercetin control (IC_50_ = 1.58 ± 0.12 µg/mL) (Figure 2B and Table 3).

*Saco* total extract already proved to be able to prevent hemolysis [7]. Considering other fruit extracts, *Saco* extracts exhibited more potential to inhibit hemolysis than aqueous extracts of strawberry fruits (*Arbutus unedo* L.) (IC_50_ = 430.00 µg/mL) [26]. Furthermore, the colored fraction displayed similar activity to methanolic extracts of grape fruits (Ruby Cabernet) (IC_50_ = 11.62 µg/mL) [39].

These results reinforce the fact that phenolic compounds, principally anthocyanins, can scavenge free radicals before they attack the membrane of erythrocytes, diminishing their concentration, and thereby preventing hemolysis [37]. These results were supported by another work concerning the anti-hemolytic effects of phenolics from honey, where it was also verified that they can capture free radicals, preventing lysis’ incident [40].

### 3.5. Effect of Sweet Cherry Extracts in Caco-2 Cell Viability

As referred above, it was already reported that sweet cherries have antidiabetic properties [7,29,30]. Hereupon, human colorectal adenocarcinoma Caco-2 cells were selected for two reasons: (i) they are a model of the intestinal epithelium, since, after differentiation, they form monolayers, that reproduce several characteristics of intestinal epithelial cells (e.g., the formation of microvillus at the apical cell surface, the tight junctions between cells, and the expression of brush-border proteins including digestive enzymes, transporters, and receptors; and (ii) it is important to note that after consumption of cherries, their compounds directly contact with intestinal epithelium [15].

**Figure 3:**
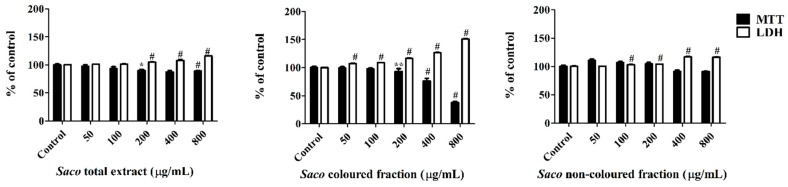
Effect of *Saco* extracts on Caco-2 cell lines viability after 24 h of exposure, assessed by 3-(4,5-dimethylthiazol-2-yl)-2,5-diphenyltetrazolium bromide (MTT) reduction and lactate dehydrogenase (LDH) leakage assays. Values show mean ± SEM of six independent assays performed in triplicate (* *p* < 0.05, ** *p* < 0.01 and ^#^ *p* < 0.0001).

In a first step, and given that phenolic compounds can exert a dose-dependent cytotoxicity, a preliminary experiment was conducted to determine the range of concentrations for which the exposure to each extract does not affect the cellular viability. As can be seen in Figure 3, Caco-2 cells proved to be more sensitive to the colored fraction when compared to the total extract and non-colored fraction, exhibiting decreases concerning their cellular viability, that ranged from 93.11% (200 µg/mL) to 73.66% (400 µg/mL) to 37.90% (800 µg/mL), and had an IC_50_ value of 667.84 ± 2.46 µg/mL. Additionally, and as expected, the more significant LDH response was also obtained using the colored fraction, mainly for the higher concentrations, such as 200, 400, and 800 µg/mL (with values of 116.5%, 126.3%, and 151.1%, respectively) (Figure 3).

The obtained data suggest that the loss of mitochondrial activity happened prior to the membrane’s damage given that the results of MTT were more expressive than those of LDH, thus discarding a necrotic process in the lowest concentrations (≤200 µg/mL) and its occurrence in the highest concentrations (400 and 800 µg/mL), which was accompanied by an elevation of LDH in the culture medium [42,43,44]. Indeed, phenolics can interfere in different steps of carcinogenesis (promotion, initiation, and progression) acting as a blocking agent or as a cancer suppressor, inhibiting the metabolic action of the pre-carcinogens, and consequently, blocking the tumor initiation [44,45]. In our work, higher concentrations of phenolics (>200 µg/mL) may induce DNA damage and necrosis in cancer cells [43,44].

The remarkable effect of the colored extract was not surprising, taking into account that the anthocyanins promoted cell death in cancer cells, triggering apoptosis through two major cell-intrinsic pathways, either by binding to the death receptors on the cell surface or by promoting the mitochondrial release of cytochrome C, inciting the depolarization of the mitochondrial membrane and the degradation of the poly[adenosine diphosphate-ribose] polymerase 1 [46]. This fact was supported by the positive correlation obtained by the total anthocyanin content and the inhibitory proliferation activity (*r* = 0.6674). These effects were also enhanced by their three OH groups on the B-ring, which are the main responsible for their biological activity [18]. Additionally, non-colored phenolics also exhibit antiproliferative effects. Particularly, Yen et al. [47] verified that many phenolic compounds, including quercetin derivatives, caffeic, chlorogenic, and p-coumaric acids, showed cytotoxicity and strong inhibitory effects on human cancer cell growth, once again due to their antioxidant capacities.

**Figure 4:**
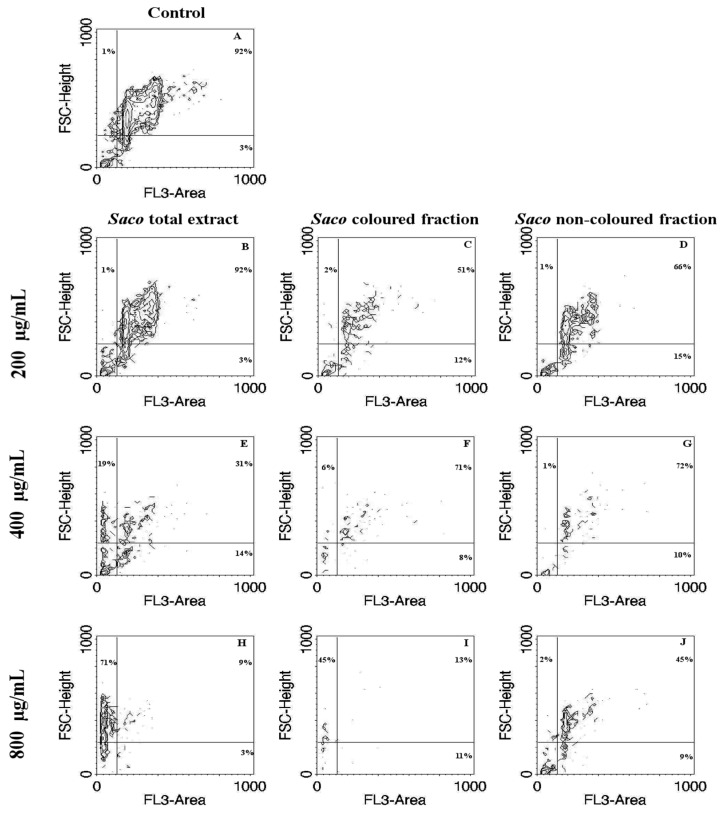
Correlation of size with Propidium Iodide (PI)-staining contour plots obtained by flow cytometric assay in Caco-2 cells treated with cherry extracts for 24 h. Untreated cells control (**A**) and cell treated with 200 (**B**–**D**), 400 (**E**–**G**), and 800 (**H**–**J**) µg/mL of each extract are shown. The extracts were *Saco* total extract (**B**,**E**,**H**), the colored extract (**C**,**F**,**I**) and the non-colored extract (**D**,**G**,**J**). The percentage of events in each quadrant is shown for a representative sample. PI-stained and normal sized events correspond to the upper right quadrant (potential live cells). PI-stained debris (potential apoptotic cells) correspond to the lower right quadrant. Left upper (potentially resulting from necrosis) and lower left quadrants are PI-negative events (potential cytoplasmic debris). FSC, forward scatter; FL3, fluorescence channel 3.

**Figure 5:**
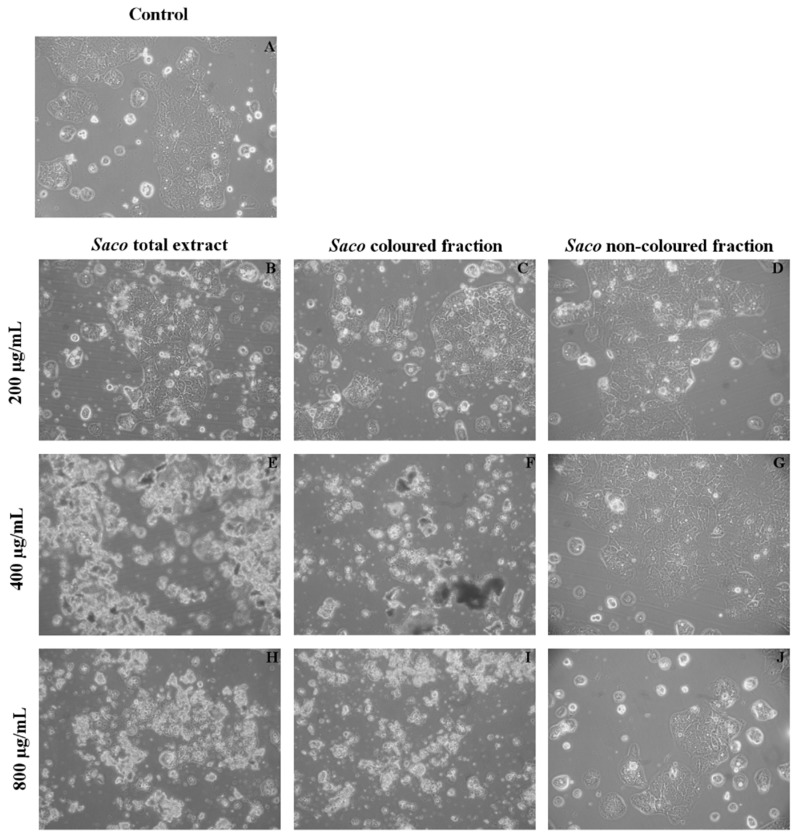
Morphological assessment of Caco-2 cells (control vs. treatment after 24 h of incubation). (**A**) corresponds to the control, (**B**,**E**,**H**) correspond to *Saco* total extract, while (**C**,**F**,**I**) to the colored fraction and (**D**,**G**,**J**) to the non-colored one, at concentrations of 200, 400 and 800 µg/mL, respectively. As expected and considering the data of Figure 4, it was observed an increase in debris as the concentration of each fraction increased.

In order to further explore the cellular mechanism of growth inhibition by cherry extracts, a morphological assessment, and an attempt of evaluation of cell cycle were performed. Apoptosis and necrosis represent two fundamental types of cell death. On the one hand, apoptosis causes several morphological and biochemical changes in cells that may take hours or days. From the morphological point of view, it is usually associated with cell shrinkage and bleb formation [47]. On the other hand, necrosis occurs suddenly, and it is characterized by mitochondrial and cellular swelling followed by plasma membrane disruption [43]. This study was performed upon incubation with the cherry extracts using the higher concentrations (200, 400, and 800 µg/mL), which were those that showed promising antiproliferative activity. The control cells positively marked with PI and gave a characteristic histogram on linear PI-fluorescence channel, but unfortunately, it was not possible to assess the cell cycle on treated cells because the number of events was not sufficient (data not shown) due to extensive cell death, as was apparent with observation under the optical microscope (Figure 4 and Figure 5). While the acquisition of controls was stopped when about 10,000 singlet events were acquired, treated cells were acquired until no more cell suspension was left, and originated a much lower event count. To visualize simultaneously morphological information (size) and PI-staining intensity of events, data were plotted on Forward Scatter (FSC)-Height vs. Fluorescence channel 3 (FL3)-Area contour plots (Figure 4). PI-stained and normal sized events decreased as the concentration increased (upright (UR) part of the plots, Figure 4B,E,H for the total extract, Figure 4C,F,I for the colored fraction, and 4D,G,J for the non-colored fraction, respectively). Furthermore, in the highest tested concentrations of the total extract and colored fraction (400 and 800 μg/mL), there was a significant increase in the amount of debris with a size bigger than usual (upper left quadrant), which may be evidence of necrotic processes (Figure 4E,H for the total extract, and Figure 4F,I for the colored fraction, respectively). Beyond that, and as expected the colored fraction was the most cytotoxic, showing the lowest event number (Figure 4I). This fact agrees with the previous result obtained through MTT and LDH assays concerning the colored fraction (Figure 3).

At the morphological observation of cultures under the microscope, a high amount of debris was observed (Figure 5) that increased with the increase of concentration (Figure 5H–J). These data were to be expected because it was already mentioned that high concentrations of phenolics can induce acute toxicity effects on cancer cells. Therefore, this work provides more support of the toxicity and pro-oxidant effects of higher levels of phenolic compounds, which are capable of affecting cell functions, such as growth and differentiation, causing probably both apoptosis and necrotic events. Specifically, it was already documented that quercetin may induce an initial shock to the cells, resulting in necrosis, followed by a reorganization of the remaining viable cells that will undergo apoptosis after prolonged treatment [43]. Furthermore, Wang et al. [44], concerning the inhibitory effect of blueberry anthocyanin extracts on melanoma cells, verified that total apoptotic cells increased gradually at concentrations of 0–800 μg/mL in a dose-dependent manner, with necrosis occurring from the concentration of 300 µg/mL.

### 3.6. Cytoprotection Assay

The second step was to evaluate the potential of each extract to protect the cells against the toxicity caused by *t*-BHP. *t*-BHP is an organic peroxide metabolized by cytochrome P450 and is widely used, being a better alternative than hydrogen peroxide in oxidation-induced stress studies since it creates more stable radical species, particularly toxic peroxyl and alkoxyl radicals that affect cell integrity and form covalent bonds with cellular molecules, promoting the death of cells [48].

**Figure 6:**
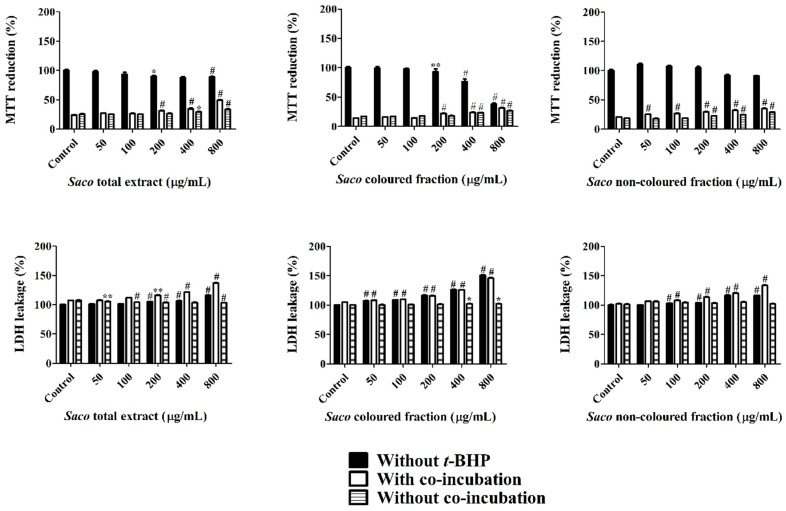
Caco-2 cellular viability assessed by 3-(4,5-dimethylthiazol-2-yl)-2,5-diphenyltetrazolium bromide (MTT) reduction and lactate dehydrogenase (LDH) leakage assays, after exposure to *Saco* extracts, with and without *tert*-butyl hydroperoxide (*t*-BHP)-induced toxicity. Cells were pre-treated with each extract for 24 h. Insulted cells were further exposed to *t*-BHP (1 mM) for 6 h. Values show mean ± SEM of six independent assays performed in triplicate (* *p* < 0.05, ** *p* < 0.01 and ^#^ *p* < 0.0001 compared to the respective controls).

Cells were treated with different concentrations of each extract for 24 h prior to *t*-BHP exposure (1 mM, 6 h). Cellular viability was again determined by MTT and LDH leakage assays. All the extracts exerted a dose-dependent protective effect in the MTT reduction and LDH leakage assays (Figure 6). Pre-treatment of Caco-2 cells with each extract was seen to significantly prevent reductions in cell viability. The total protection, when compared to stressed control cells, was almost achieved at the concentrations of 50 and 200 µg/mL with and without co-incubation with *t*-BHP for the total extract and non-colored fraction. While for the colored fraction, this one was achieved at concentrations of 50 and 100 µg/mL with and without co-incubation, respectively (Figure 6). Relative to the assay with co-incubation, there were observed increments of viability between 2.89% and 25.45% for the total extract, 4.98% and 14.46% for the non-colored fraction, and between 1.73% and 17.22% for the colored fraction (Figure 6). Concerning the obtained data without co-incubation, lower values of protection were observed, ranging from 25.00 to 33.58% for the total extract, 18.15 to 28.96% for the non-colored fraction, and 16.90% to 27.02% for the colored fraction (Figure 6). This outcome indicates that the presence of phenolics in the medium, even outside the cells, also offers protection against cellular damage [44].

In both assays, the highest protection was verified with the total extract at the concentration of 800 µg/mL, which is another support about the simultaneous interaction and synergy between colored and non-colored phenolic compounds [44].

Once again and given the oxidative-stress induced by *t*-BHP, it was verified that there was an increase in LDH leakage, most notorious in the assay without co-incubation, revealing that the permanence of phenolics in the medium increases the protective effects on cells. Our results are in agreement with the work of Bedoya-Ramírez et al. [49], who reported that 500 µg/mL of Colombian coffee can increase cell viability between 34% and 45% evaluated by MTT assay.

The phenolic composition of each extract is described in Table 1 and Table 2. In fact, phenolics are greatly responsible for the observed antioxidant protective effects. As is already known, mitochondria are very susceptible to oxidative stress induced by oxygen species generated continuously in these organelles in the course of oxidative stress [48]. Phenolics, essentially due to their hydroxyl and carboxyl groups, exhibit promising and strong antioxidant activities being capable of capturing these radicals and chelating metals before they attack the mitochondrial membrane [42]. Particularly, 50 µM of quercetin derivatives already showed similar protective effects at preventing Caco-2 damage induced by *t*-BHP [50].

## 4. Conclusions

Considering the current interest in cherries, the present study describes the phenolic constitution and health-promoting properties of three extracts from *Saco* sweet cherry. A total of 22 phenolic compounds, including 17 non-colored phenolics and 5 anthocyanins, were identified, hydroxycinnamic acids and cyanidin-3-*O*-rutinoside being the main ones. In relation to biological potential, in a general way, the total extract proved to be the most active given the interactions established between both colored and non-colored phenolic compounds. Even so, all extracts revealed facility to scavenge free radical species and to inhibit the *α*-glucosidase intestinal enzyme. Concerning human erythrocytes protection, the colored fraction was the most effective, mainly due to the presence of many OH groups in anthocyanins, which increases the capacity of scavenging ROO^•^ species. On the other hand, and relative to the Caco-2 cell study, the highest tested concentrations of each extract showed the ability to inhibit the proliferation of Caco-2 cells mainly due to their antioxidant properties. Additionally, both fractions and the total extract exerted a cytoprotective effect against oxidative stress induced by *t*-BHP in Caco-2, improving cell viability. Once again, this protection was closely linked to the capacity of phenolics to pass through the cellular membrane and act as direct antioxidants, capturing free radicals and chelating metals. Our results suggest that phenolic compounds are a major factor correlated with health benefits. However, our toxicity results justify concerns regarding the consumption of high doses that become unsafe and harmful to human health. Therefore, further animal and human trials are needed to unravel ensure safe dosage of sweet cherry fractions and to incite their use to enrich food and nutraceutical or pharmaceutical products.

## Figures and Tables

**Table 1:** Non-colored phenolic contents of *Saco* sweet cherry extracts (μg/g of dried extract).

Non-Colored Phenolics	Total Extract	Non-Colored Fraction
Hydroxybenzoic acid derivative 1	1337.85 ± 68.16	1839.54 ± 5.09 ^a^
Hydroxycinnamic acid derivative 1	494.32 ± 51.66	679.69 ± 71.03
Hydroxycinnamic acid derivative 2	143.45 ± 21.30	197.24 ± 29.28
Hydroxybenzoic acid derivative 2	25.08 ± 0.92	34.48 ± 1.27
3-*O*-Caffeoylquinic acid	1482.97 ± 54.15	2039.09 ± 74.45 ^a^
*ρ*-Coumaric acid derivative 1	50.02 ± 0.55	68.78 ± 0.76
*ρ*-Coumaroylquinic acid	nq	nq
Hydroxycinnamic acid derivative 3	372.26 ± 35.99	511.86 ± 49.48
5-*O*-Caffeoylquinic acid	734.38 ± 44.86	1009.77 ± 61.68 ^a^
Hydroxycinnamic acid derivative 4	2835.87 ± 143.08	3899.33 ± 196.73 ^a^
Caffeic acid	1263.49 ± 98.92	1737.30 ± 136.01 ^a^
*p*-Coumaric acid derivative 2	528.74 ± 19.83	727.02 ± 27.26
Hydroxycinnamic acid derivative 5	704.96 ± 97.52	969.31 ± 134.08 ^a^
Hydroxycinnamic acid derivative 6	196.75 ± 16.19	270.53 ± 22.26
*p*-Coumaric acid	21.07 ± 1.64	28.96 ± 2.26
Hydroxycinnamic acid derivative 7	666.97 ± 67.02	917.089 ± 92.15 ^a^
Hydroxycinnamic acid derivative 8	175.97 ± 16.59	241.95 ± 22.80
Quercetin-3-*O*-glucoside	nq	nq
Kaempferol-3-*O*-rutinoside	nq	nq
Quercetin	35.58 ± 3.73	48.93 ± 5.13
Σ	11,069.73	15,220.88

Values are expressed as mean ± standard deviation of three assays. ∑, sum of the determined non-colored phenolics; nq, not quantified. ^a^ Significant result (*p* < 0.05) is indicated as vs. total extract.

**Table 2:** Anthocyanin contents of *Saco* sweet cherry extracts (μg/g of dried extract).

Anthocyanins	Spectra of Absorption (nm)	Total Extract	Coloured Fraction
Unknown 1	500	2.99 ± 0.24	nd
Cyanidin-3-*O*-glucoside	500	193.48 ± 0.54	3427.93 ± 4.39 ^a^
Cyanidin-3-*O*-rutinoside	500	3865.64 ± 2.95	15656.18 ± 25.71 ^a^
Unknown 2	500	341.16 ± 2.82	nq
Pelargonidin-3-*O*-rutinoside	500	337.464 ± 20.19	130.39 ± 1.22 ^a^
Peonidin-3-*O*-rutinoside	500	nq	nd
Σ		4740.73	19,214.50

Values are expressed as mean ± standard deviation of three assays. ∑, sum of the determined anthocyanins; nd; not detectable; nq, not quantified. ^a^ Significant result (*p* < 0.05) is indicated as vs. total extract.

**Table 3:** IC_25_ and IC_50_ (μg/mL) values found in the antioxidant activity, α-glucosidase, hemoglobin oxidation, and hemolysis assays of *Saco* sweet cherry extracts.

Extract	DPPH^•^	^•^NO	O_2_^•−^	*α*-Glucosidase	Hemoglobin Oxidation	Hemolysis
Total extract	21.88 ± 0.32	33.72 ± 0.89	41.68 ± 0.72	53.15 ± 1.32	34.29 ± 0.88	28.71 ± 0.73
Colored fraction	31.39 ± 0.60 ^a^	47.44 ± 0.67 ^a^	16.58 ± 0.27 (IC_25_)	142.02 ± 1.17 ^a^	33.86 ± 0.70	9.44 ± 0.48
Non-colored fraction	210.86 ± 0.86 ^a,b^	167.96 ± 0.92 ^a,b^	69.40 ± 1.22 ^a,b^	456.19 ± 3.74 (IC_25_)	155.13 ± 1.45 ^a,b^	48.31 ± 1.07 ^a,b^

Values are expressed as mean ± standard deviation of three assays concerning the antioxidant capacity against 1,1-diphenyl-2-picrylhydrazyl, nitric oxide and superoxide radicals (DPPH^•^, ^•^NO and O_2_^•−^, respectively), *α*-glucosidase inhibitory activity, hemoglobin oxidation and hemolysis. IC_25_: 25% inhibitory concentration. ^a^ Significant result (*p* < 0.05) is indicated as vs. total extract. ^b^ *p* < 0.05 is indicated as vs. colored fraction.
